# Supplementary figures and images for: Unveiling the endogenous CRISPR-Cas system in Pseudomonas aeruginosa PAO1
Source: PLoS One. 2024 Dec 31;19(12):e0312783. doi: 10.1371/journal.pone.0312783 (PMC11687729; doi:10.1371/journal.pone.0312783)

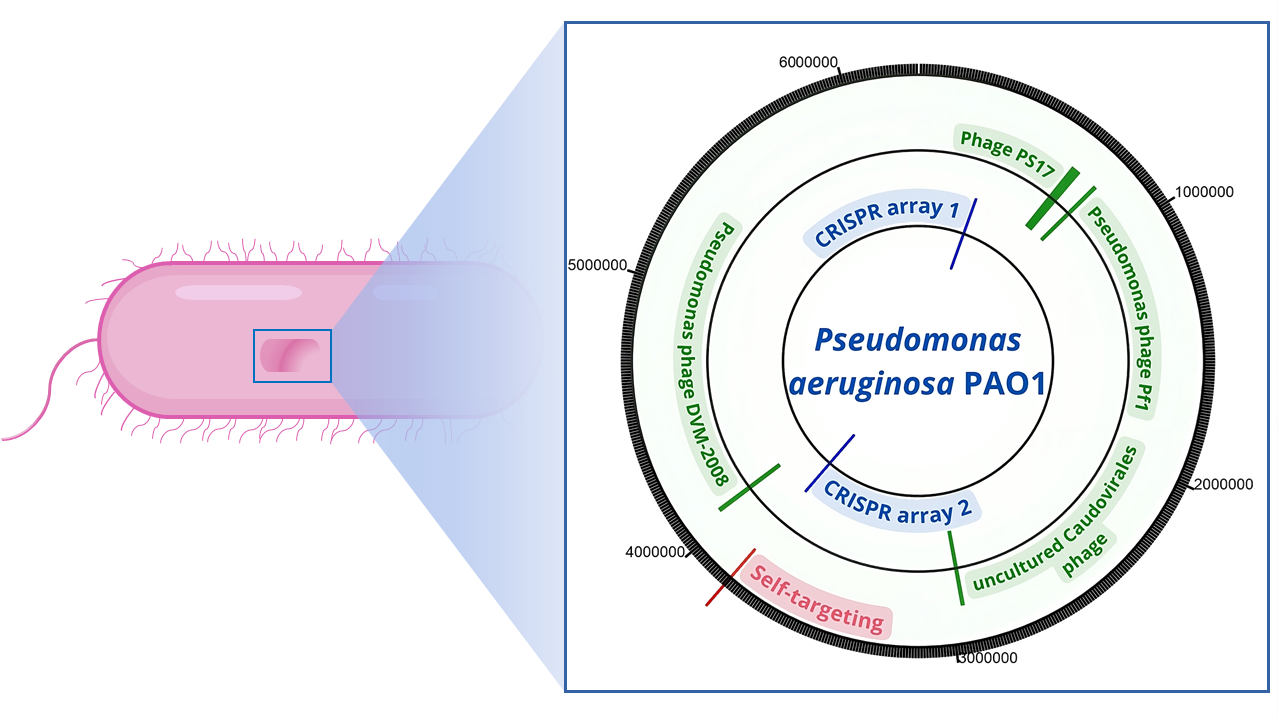

Supplement: S1 Graphical abstract — (TIF) [file pone.0312783.s005.tif]
